# Exploring Relationships Between Multimodal Lifestyle Factors, Inflammatory Biomarkers, and Pain Processing in Women with Fibromyalgia: An Exploratory Pilot Study

**DOI:** 10.3390/nu18213507

**Published:** 2026-10-25

**Authors:** Ömer Elma, Christopher Long, Amber Brown, Jamie Dresse, Karen Yendole, Carol Clark, Jonathan Williams, Rebecca A. Neal, Nils Runge, Emma Mosley, Sevilay Tümkaya Yılmaz, Sumanto Haldar

**Affiliations:** 1 School of Allied Health and Exercise Sciences, Faculty of Health, Environment and Medical Sciences, Bournemouth University, Bournemouth BH8 8GP, UK; cjlong@bournemouth.ac.uk (C.L.); cclark@bournemouth.ac.uk (C.C.); jwilliams@bournemouth.ac.uk (J.W.); rneal@bournemouth.ac.uk (R.A.N.); mosleye@bournemouth.ac.uk (E.M.); shaldar@bournemouth.ac.uk (S.H.); 2 Pain Science Research Group (PSRG), Centre for Wellbeing and Long-Term Health, Bournemouth University, Bournemouth BH8 8GP, UK; sevilay.tumkaya.yilmaz@vub.be; 3 Pain in Motion Research Group (PAIN), Department of Physiotherapy, Human Physiology and Anatomy, Faculty of Physical Education and Physiotherapy, Vrije Universiteit Brussel, Brussels 1090, Belgium; nils.arno.andreas.runge@vub.be; 4 VItal Signs and PERformance Monitoring (VIPER), LIFE Department, Royal Military Academy, 1000 Brussels, Belgium

**Keywords:** fibromyalgia, chronic pain, lifestyle factors, stress, diet, systemic inflammation, quantitative sensory testing

## Abstract

**Background:** Fibromyalgia is a chronic pain condition characterised by altered pain processing, widespread pain, and symptoms including sleep disturbance, fatigue, and psychological distress. Emerging evidence suggests that modifiable multimodal lifestyle factors may influence inflammation and pain mechanisms; however, the relationships between multimodal lifestyle factors, systemic inflammatory biomarkers, and pain processing remain poorly understood. In this study we aimed to investigate the associations between lifestyle factors, inflammatory biomarkers, and experimental and clinical pain outcomes in women with fibromyalgia. **Methods:** Women with fibromyalgia were invited to participate in this exploratory cross-sectional study. Lifestyle factors included diet, perceived stress, sleep, physical activity, heart rate variability, and BMI. Plasma IL-6, IL-10, and TNF-α were measured. Pain outcomes included quantitative sensory testing and patient-reported outcome measures. Spearman’s rank correlations and multiple linear regression analyses adjusted for age and disease duration were performed. Bootstrap 95% confidence intervals were calculated for all estimates, and False Discovery Rate (FDR) corrections using the Benjamini–Hochberg procedure were applied as a sensitivity analysis. **Results:** Twenty patients were included. Correlation analyses demonstrated that higher perceived stress was strongly associated with greater fibromyalgia impact (r = 0.68, *p* < 0.001, n = 20), central sensitization (r = 0.67, *p* < 0.001, n = 20), and current pain intensity (r = 0.64, *p* = 0.002, n = 20). A more pro-inflammatory diet was positively associated with IL-6 (r = 0.46, *p* = 0.04, n = 20) and IL-10 (r = 0.47, *p* = 0.10, n = 13), while higher TNF-α concentrations were associated with lower pressure pain thresholds (overall; r = −0.55, *p* = 0.021, n = 19; tibialis: r = −0.57, *p* = 0.010, n = 19). Following adjustment for age and disease duration, associations between stress and fibromyalgia impact, central sensitization, and current and worst pain intensity, together with associations between pro-inflammatory diet and IL-6 and IL-10, and between BMI and IL-10 showed similar directions and effect estimates. However, following FDR adjustment as a sensitivity analysis, no associations retained statistical significance. **Conclusions:** This study suggests that different modifiable lifestyle factors may be associated with distinct aspects of pain and inflammatory processes in women with fibromyalgia. Perceived stress showed the most consistent associations with pain-related outcomes, while dietary inflammatory potential showed preliminary associations with inflammatory biomarkers that need further investigation.

## 1. Introduction

Fibromyalgia is a debilitating chronic condition characterised by widespread musculoskeletal pain, fatigue, sleep disturbance, cognitive dysfunction, and a range of somatic symptoms that substantially reduce quality of life and functional capacity [1]. It affects approximately 2–3% of the global population and is considerably more prevalent in women than men [1]. Although its pathophysiology remains incompletely understood, current evidence suggests that fibromyalgia results from complex interactions between altered central pain processing, neuroendocrine dysfunction, autonomic dysregulation, gut dysbiosis, and low-grade systemic inflammation [2]. Consequently, individuals with fibromyalgia commonly exhibit pain amplification, impaired endogenous pain inhibition, enhanced pain facilitation, and widespread pain hypersensitivity [3]. Given its multifactorial nature, there is increasing recognition that fibromyalgia requires a biopsychosocial management approach that incorporates multimodal, modifiable lifestyle factors [4].

Modifiable lifestyle factors have recently gained considerable attention in chronic pain research because they represent potentially sustainable, non-pharmacological targets for personalised and support self-management [5,6]. Lifestyle factors including dietary habits, sleep, psychological stress, physical activity, obesity, smoking, and alcohol consumption have all been suggested to influence fibromyalgia symptoms [4]. For example, higher body mass index (BMI), lower physical activity levels, and smoking have been associated with an increased risk of self-reported fibromyalgia [7]. Psychological stress and poor sleep quality have been linked to dysregulation of the autonomic nervous system, central sensitization, impaired pain modulation, and greater symptom severity [8]. Similarly, obesity and physical inactivity may contribute to persistent pain through metabolic dysfunction, reduced physical capacity, and increased inflammatory activity [9]. Among these lifestyle factors, diet has emerged as a particularly promising area of investigation because of its potential to influence the gut-microbiome, systemic inflammation, and neuroimmune responses [10]. More pro-inflammatory dietary patterns have been associated with elevated inflammatory biomarkers and poorer health outcomes across several chronic diseases [11,12]. Furthermore, a large-scale population study demonstrated that higher adherence to healthy and plant-based dietary indices is associated with significantly lower odds of experiencing chronic pain conditions including fibromyalgia [13]. In fibromyalgia patients, evidence also suggests that dietary interventions, including vegetarian, vegan, and plant-based dietary patterns, may improve pain and quality of life [14]. However, the existing evidence remains fragmented, with most studies examining individual lifestyle factors or dietary patterns in isolation and relying predominantly on self-reported measures.

Increasing evidence suggests that lifestyle behaviours do not influence pain independently but rather through interconnected biological pathways [15]. Factors such as diet, obesity, psychological stress, sleep, physical activity, smoking, and alcohol consumption may alter neuroendocrine function, autonomic regulation, immune responses, and the gut–brain axis, collectively influencing pain processing [16]. For example, gastrointestinal comorbidities, particularly irritable bowel syndrome, are common in fibromyalgia, and gut microbial dysbiosis has been proposed as one mechanism linking lifestyle behaviours with altered immune function, neuroimmune responses, and pain processing [17,18]. These pathways may eventually contribute to low-grade systemic inflammation, which has increasingly been recognised as a potential mechanism underlying fibromyalgia. Although fibromyalgia is not considered a classical inflammatory disease, altered circulating cytokines and central neuroinflammatory processes have been associated with central sensitization, pain processing, and symptom severity [19].

Taken all together, previous studies have generally examined individual lifestyle factors, inflammatory biomarkers, or clinical outcomes separately, leaving the interplay between these domains poorly understood. Examining these domains together may provide preliminary evidence to identify clinically relevant targets for personalised lifestyle interventions and inform future multidisciplinary management strategies. Therefore, this exploratory cross-sectional pilot study mainly aimed to investigate the relationships between multimodal lifestyle factors, systemic inflammatory biomarkers, and pain-related outcomes in women with fibromyalgia. The specific objectives were to explore the associations between lifestyle factors, systemic inflammatory biomarkers, and pain-related outcomes and determine whether the strongest observed associations remained after adjustment for clinically relevant covariates using exploratory regression analyses; evaluate the feasibility of implementing the multimodal assessment protocol; and obtain preliminary estimates of effect sizes and variability to inform sample size calculations for future adequately powered studies.

## 2. Materials and Methods

### 2.1. Study Design, Setting, and Ethics

This exploratory cross-sectional pilot study was conducted at the Human Performance Laboratory, Bournemouth University, United Kingdom. Ethical approval was obtained from the Bournemouth University Research Ethics Committee (Ethics ID: 61104), and the study was retrospectively registered on ClinicalTrials.gov (ID: NCT07074275) on 30 June 2025, prior to completion of the primary data collection. All participants provided written informed consent prior to participation. The study was conducted in compliance with the Declaration of Helsinki, with primary data collection conducted between May and August 2025.

### 2.2. Sample Size

Given the exploratory nature of this pilot study, a formal a priori sample size calculation was not performed. In accordance with recommendations that sample size in pilot studies should be determined according to the aims and intended use of the pilot data [20], a sample of 20 participants was considered appropriate for obtaining preliminary estimates of variability and potential associations between multimodal lifestyle factors, inflammatory biomarkers, and pain-related outcomes and for informing the design and sample size requirements of future adequately powered studies.

### 2.3. Participants and Recruitment

Female native English-speaking adults aged between 18 and 65 years old with a clinician-confirmed diagnosis of fibromyalgia according to the 2016 American College of Rheumatology diagnostic criteria were eligible for inclusion [21]. Participants were also required to have chronic pain, defined as pain persisting for at least three months and occurring on at least three days per week. Participants were excluded if they had any other systemic inflammatory or metabolic condition (e.g., diabetes mellitus, arthritis, lupus, or other autoimmune disorders), a history of cancer within the previous five years, were receiving anti-inflammatory and immunosuppressive medications (e.g., corticosteroids, chemotherapy, biologics), or were pregnant or had been pregnant within the previous 12 months. Participants were recruited through social media advertisements, online fibromyalgia support groups, and public advertisements. Recruitment was open to all eligible members of the community.

### 2.4. Study Procedures

**Figure 1:**
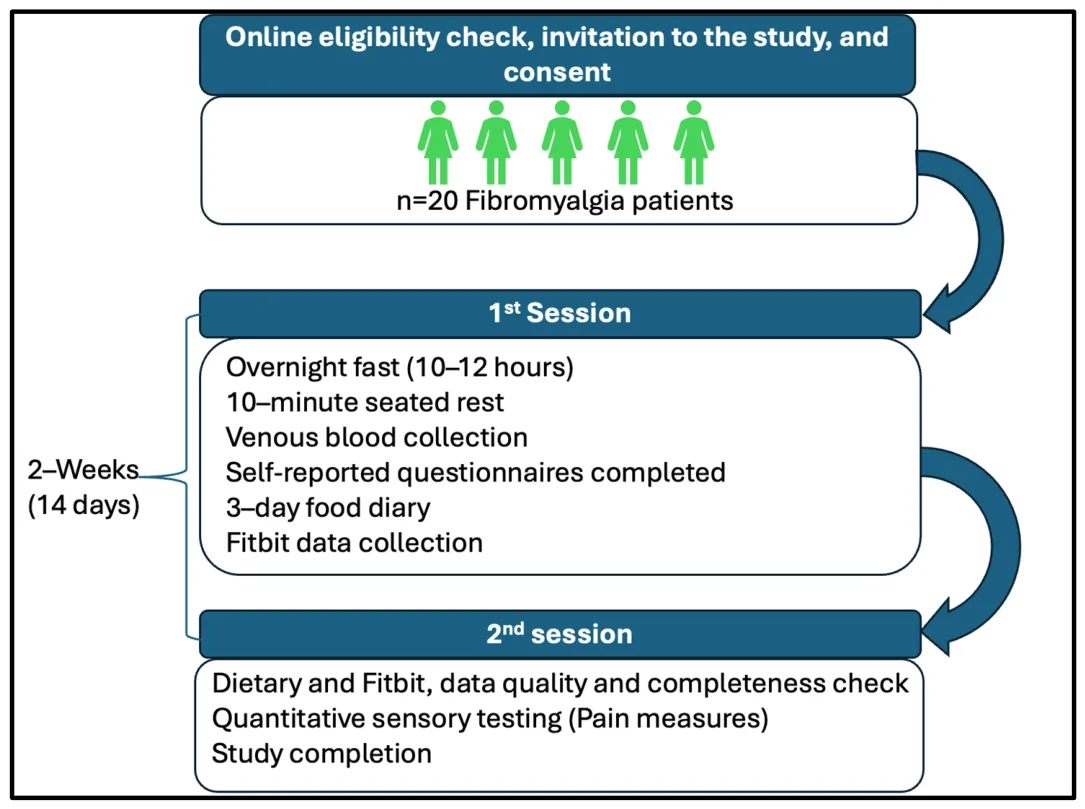
Study flow diagram illustrating participant recruitment and data collection procedures.

Following initial contact, potential participants completed an online eligibility screening questionnaire. Eligible participants were invited to attend two assessment sessions separated by two weeks. An overview of the participant recruitment and study procedures is presented in Figure 1.

The 1st session was scheduled between 08:00 and 10:00 h following an overnight fast of approximately 10–12 h. After written informed consent and a minimum of 10-min seated rest period, venous blood samples were collected by a phlebotomy trained and certified practitioner (S.H.). Participants subsequently completed the self-reported questionnaires and received detailed instructions for completing a 3-day food diary. Finally, participants were given a Fitbit Charge 6 (Google LLC, USA) and instructed on its use, charging procedures, and synchronisation with the accompanying mobile application. Participants were asked to wear the device continuously for the following 14 days, removing it only when necessary for charging.

At the 2nd session after 14 days, Fitbit data were downloaded from participants’ Fitbit accounts and checked for completeness. Food diaries were reviewed for completeness by registered nutritionists (A.B. and J.D.) to ensure that the records were accurately completed. Participants then underwent quantitative sensory testing (QST) by an experienced pain researcher (Ö.E.).

### 2.5. Outcome Measures

#### 2.5.1. Experimental Pain Measures (Quantitative Sensory Testing)

##### Pressure Pain Threshold

Pressure pain threshold (PPT) was assessed using a digital pressure algometer (FPX-25, Wagner Instruments, Greenwich, CT, USA). Pressure was applied at a constant rate of approximately 0.5 kg/cm^2^ per second [11]. Participants were instructed to indicate the point at which the sensation first became painful. Before testing commenced, participants were familiarised with the procedure using the volar aspect of the non-dominant forearm. PPT was then assessed bilaterally over the mid-belly of the upper trapezius muscle (midway between the C7 spinous process and the acromion) and the tibialis anterior muscle (approximately 5 cm distal to the tibial tuberosity), at anatomical sites commonly used in fibromyalgia and other chronic pain conditions [22]. Three measurements were obtained from each site with a 1-min rest period between consecutive PPT measurements to minimise temporal sensitisation. The mean value from the three measurements was calculated for each site [11]. Average PPT values for the trapezius and tibialis anterior were calculated, and an overall PPT score was derived as the mean PPT across all assessed body sites for statistical analyses. A 5-min rest period was provided before proceeding to the temporal summation assessment.

##### Temporal Summation

Temporal summation (TS) was used to assess endogenous pain facilitation. Ten consecutive pressure stimuli were applied over the upper trapezius using each participant’s mean trapezius PPT. The TS score was calculated as the difference between the pain rating recorded following the tenth and first stimulus, with higher values indicating greater pain facilitation [11]. Following completion of the TS assessment, participants rested for 5 min before commencing the conditioned pain modulation protocol.

##### Conditioned Pain Modulation

Conditioned pain modulation (CPM) was assessed using an ischaemic cuff paradigm. A blood pressure cuff (Welch Allyn, NY, USA) was inflated around the upper arm to either 200 mmHg or until participants reported pain intensity of 6/10 on a visual analogue scale. The cuff remained inflated for a maximum of 10 min. PPT was reassessed during cuff inflation at the dominant trapezius site. CPM was calculated as the difference between PPT measured before and during cuff inflation, with larger values indicating greater endogenous pain inhibitory capacity [23].

#### 2.5.2. Inflammatory Biomarkers

Venous blood samples were collected using butterfly needles into 10 mL EDTA tubes following an overnight fast (minimum 10 h) and a 10-min seated rest period following arrival at the study centre. Samples were centrifuged at 1500× *g* at 4 °C for 15 min immediately following collection. Plasma was aliquoted into labelled 1.5 mL Eppendorf tubes and stored at −80 °C until analysis.

Plasma concentrations of interleukin-6 (IL-6-ab46042 high sensitivity human kit), interleukin-10 (IL-10-ab46059 high sensitivity human kit), and tumour necrosis factor-alpha (TNF-α-ab46087 human kit) were quantified using commercially available enzyme-linked immunosorbent assay (ELISA) kits (Abcam, Cambridge, UK). All samples were analysed in duplicate, with one control sample included for each assay. Standard curves were generated using concentrations ranging from 1.56 to 50 pg/mL for IL-6 and IL-10, and from 12.5 to 400 pg/mL for TNF-α. Following the manufacturer’s protocols, absorbance was measured using a microplate reader, and cytokine concentrations were calculated from the corresponding standard curves. The manufacturer-reported intra-assay and inter-assay coefficients of variation were 4.4% and 9.1% for IL-6, 7.8% and 10.2% for IL-10, and 3.3% and 9.0% for TNF-α, respectively.

Plasma samples were available for all 20 participants, and no participant-level clinical or behavioural data were missing. However, for IL-10, seven of 20 samples were below the lower end of the standard curve and therefore below the assay’s quantitative range (<1.56 pg/mL). These samples were not assigned numerical values, resulting in n = 13 for IL-10 analyses. One TNF-α sample was also below the assay’s quantitative range and excluded from quantitative analyses, resulting in n = 19.

#### 2.5.3. Dietary Assessment

Habitual dietary intake was assessed using a 3-day food diary consisting of two weekdays and one weekend day. Participants were instructed to record their usual dietary intake and avoid selecting atypical days, such as fasting days or special occasions. Detailed instructions were provided regarding meal timing, food preparation methods, brand names where appropriate, portion sizes, and household measures. Dietary records were analysed using Nutritics dietary analysis software (Nutritics Ltd., version 6.15, Dublin, Ireland), providing mean daily intake estimates of energy, macro- and micronutrients as well as food groups intake.

The inflammatory potential of habitual dietary intake was assessed using the energy-adjusted Dietary Inflammatory Index (EDII), an adaptation of the Dietary Inflammatory Index (DII) developed by Shivappa et al. [24]. The DII was developed from a comprehensive review of approximately 6500 scientific publications investigating the effects of dietary constituents on systemic inflammatory biomarkers, including C-reactive protein (CRP), tumour necrosis factor-alpha (TNF-α), and interleukins IL-1β, IL-4, IL-6, and IL-10. The original DII incorporates 45 dietary parameters; each assigned an inflammatory effect score based on the published literature [24].

Twenty-nine available dietary components were included in the calculation: protein, total fat, carbohydrate, sugars, alcohol, caffeine, cholesterol, fibre, trans fatty acids, saturated fatty acids, monounsaturated fatty acids, polyunsaturated fatty acids, omega-3 polyunsaturated fatty acids, omega-6 polyunsaturated fatty acids, vitamins A, C, D, B1, B2, B3, B6, B9 and B12, beta-carotene, iron, magnesium, zinc, selenium, and total energy intake. The EDII was calculated using the density method. Each individual nutrient component was scaled per 1000 kcal. Then, individual nutrient intakes were standardised using the adjusted global mean intake and standard deviation, converted to centred percentile values, multiplied by their respective inflammatory effect scores, and summed to derive the energy-adjusted dietary inflammatory score [24]. To account for differences in total energy intake between participants, an EDII was calculated by excluding total energy intake (kcal) from the final score, thereby reducing potential confounding by overall caloric intake [24,25]. Higher (more positive) EDII scores indicate a more pro-inflammatory dietary pattern, whereas lower (more negative) scores represent a more anti-inflammatory dietary pattern.

Ultra-processed food (UPF) intake was assessed using the NOVA food classification system [26]. All foods and beverages recorded in the dietary diaries were classified according to their degree of processing. For each recording day, the percentage of total energy intake derived from ultra-processed foods was calculated, and the mean percentage across the three recording days was used as the final UPF score. UPF values ranged from 0% to 100%, with higher scores indicating greater consumption of ultra-processed foods [26]. NOVA classification for the ultra-processed category is provided in Supplementary Table S1.

#### 2.5.4. Wearable Technology

Participants wore a Fitbit Charge 6 continuously for 14 days. Mean daily step count as an indication of the overall physical activity level, mean nightly sleep duration, and heart rate variability (HRV) quantified as the root mean of successive differences (RMSSD) were extracted from the device and used for statistical analyses.

#### 2.5.5. Self-Reported Outcome Measures

Pain intensity was assessed using the Brief Pain Inventory (BPI), which evaluates the worst, least, average and current pain intensity during the previous 24 h and in general, together with pain interference across seven domains of daily living [27]. In this study only the pain intensity section was used.

Central sensitisation symptoms were measured using Section A of the Central Sensitization Inventory (CSI), consisting of 25 items scored from 0 to 100, with higher scores indicating greater central sensitisation symptoms [28].

Fibromyalgia-related health status was assessed using the Fibromyalgia Impact Questionnaire (FIQ). The questionnaire comprises ten domains named physical impairment, feel good, work missed, do work, pain, fatigue, rested, stiffness, anxiety, and depression. Total scores range from 0 to 100, with higher scores indicating greater disease impact [29].

Perceived psychological stress was measured using the 10-item Perceived Stress Scale (PSS-10). Total scores range from 0 to 40, with higher scores representing greater perceived stress [30].

Subjective sleep quality was assessed using the Pittsburgh Sleep Quality Index (PSQI). The questionnaire evaluates seven components of sleep quality focussing on subjective sleep quality, sleep duration, latency, efficiency, disturbance, medication, and dysfunction, producing a global score ranging from 0 to 21, where higher scores indicate poorer sleep quality [31].

The smoking status of the participants was assessed with a three-layer question which consisted of answers of “current smoker”, “previous smoker”, and “never”, and responses were coded from 1 to 3 respectively.

Alcohol consumption was recorded according to habitual drinking frequency using seven categories including “daily or almost daily”, “three or four times a week”, “once or twice a week”, “one to three times a month”, “special occasions”, “previous”, and “never”. Then participants responses were coded from 1 to 7, respectively.

#### 2.5.6. Body Mass Index (BMI)

Body weight was measured in kilograms (kg) using a calibrated SECA digital weighing scale, while body height was measured in centimetres (cm) using a SECA stadiometer. BMI was subsequently calculated as body weight (kg) divided by height squared (m^2^).

### 2.6. Data Analysis

All statistical analyses were performed using Python (version 3.9.12). Normality of continuous variables was assessed using the Shapiro–Wilk test, with results presented in Supplementary Table S2, together with visual inspection of quantile–quantile (Q-Q) plots in Supplementary Figure S1. Descriptive statistics were used to summarise participant characteristics and study variables. Continuous variables are presented as the median and interquartile range (IQR), while categorical variables are presented as frequencies and percentages (n, %).

Given the exploratory nature of the study, the small sample size (n = 20), the inclusion of ordinal variables, and the non-normal distribution of several continuous variables, associations between lifestyle factors, inflammatory biomarkers, and pain-related outcomes were examined using Spearman’s rank correlation coefficient (r). Categorical variables with multiple response categories, including smoking status and alcohol consumption, were not included in the Spearman correlation analyses because their response categories did not necessarily represent a meaningful underlying ordinal scale, and numerical coding could therefore impose an artificial ranking between categories. Smoking status and alcohol consumption were consequently summarised descriptively.

To quantify the uncertainty around the estimated Spearman correlations, non-parametric bootstrap analyses with 10,000 resamples were performed, and 95% confidence intervals calculated for each correlation coefficient. The complete bootstrap results are presented in Supplementary Tables S3–S5.

The magnitude of correlations was interpreted as very weak/negligible (|r| = 0.00–0.19), weak (|r| = 0.20–0.39), moderate (|r| = 0.40–0.59), strong (|r| = 0.60–0.79), or very strong (|r| = 0.80–1.00), based on the absolute value of the correlation coefficient [32]. Correlation analyses were presented as three separate domains: pain versus lifestyle factors outcomes, pain versus inflammatory biomarkers, and lifestyle versus inflammatory biomarkers outcomes. Clustered heatmaps were generated to facilitate visual interpretation of the correlation matrices.

Exploratory multiple linear regression analyses were subsequently performed for the correlations demonstrating moderate-to-strong effect sizes (|r| ≥ 0.40). Regression models were adjusted for prespecified covariates: age and disease duration. To minimise overfitting, the number of predictors included in each model was intentionally restricted in view of the pilot sample size. Categorical variables with multiple response categories, including smoking status and alcohol consumption, were also not entered into the regression models due to limited statistical power and the risk of model overfitting.

### False Discovery Rate Sensitivity Analysis

Statistical significance was set at a two-sided *p*-value < 0.05. Given the exploratory, hypothesis-generating nature of this exploratory study, False Discovery Rate (FDR) corrections using the Benjamini–Hochberg procedure were conducted as a sensitivity analysis to evaluate the robustness of associations, with complete FDR-adjusted results reported in Supplementary Tables S3–S5. To identify informative patterns among these preliminary findings, the main text focused on effect magnitude, direction of associations, uncorrected *p*-values, and bootstrap confidence intervals, while FDR-adjusted *p*-values were considered in a sensitivity analysis.

## 3. Results

### 3.1. Participant Characteristics

**Figure 2:**
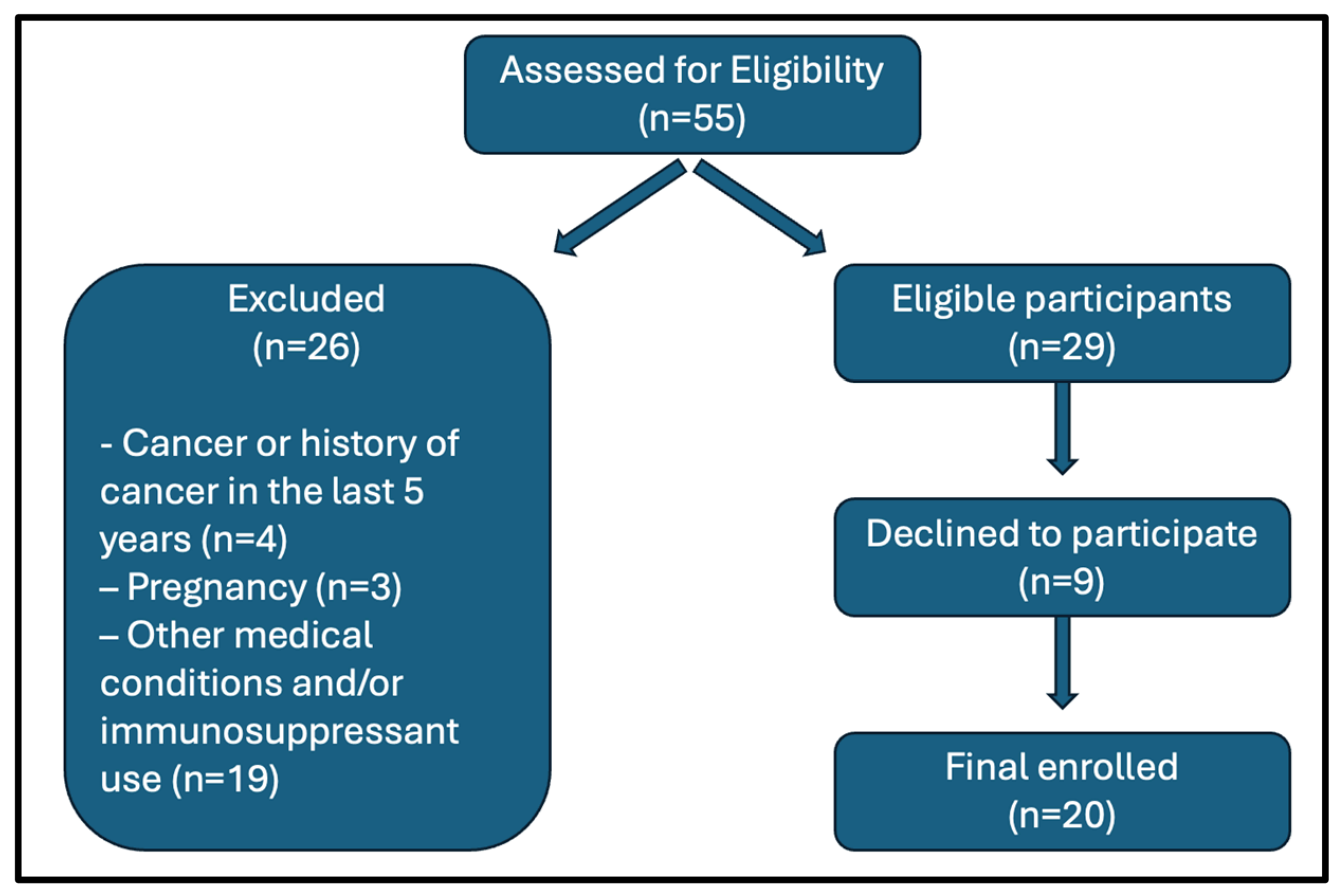
Clustered Spearman correlation heatmap illustrating relationships between lifestyle factors and pain outcomes in women with fibromyalgia.

Twenty female participants with clinically diagnosed fibromyalgia were included in the study out of 55 screened individuals (Figure 2).

There were no dropouts as all participants attended both sessions. All 20 participants fully completed all self-reported questionnaires and dietary diaries, wore the Fitbit tracker for the entire monitoring period, provided blood samples, and underwent quantitative sensory testing. However, due to cytokine concentrations falling below the quantitative range of the assays, quantifiable concentrations were available for 13 participants for IL-10 and 19 participants for TNF-α. The median age was 48 years (IQR: 35–51 years), and the median time since diagnosis was 66 months (IQR: 18–90 months). Detailed participant characteristics, lifestyle measures, pain-related outcomes, and inflammatory biomarkers are summarised in Table 1.

The median BMI was 28.1 kg/m^2^ (IQR: 22.9–39.6), indicating that the cohort was, on average, within the overweight range, with the upper quartile extending into the obesity range. The median EDII score was 3.32 (IQR: 3.1.28–4.03), which reflects a predominantly pro-inflammatory dietary pattern. Ultra-processed foods accounted for 50.0% (IQR: 37.8–60.6) of total mean daily energy intake. Half of the participants had never smoked (50.0%), 40.0% were previous smokers, and 10.0% were current smokers. Alcohol consumption varied across the cohort, with most participants reporting consumption once or twice a week (25.0%), one to three times per month (20.0%), or on special occasions (20.0%). Objective physical activity and sleep monitoring via Fitbit showed a median of 6140 steps per day (IQR: 4366–9343 steps) and 7.5 h of sleep per night (IQR: 6.7–7.7 h).

The overall median of PPT was 4.35 kg/cm^2^ (IQR: 3.27–6.70). Median PPT was lower over the upper trapezius (4.05 kg/cm^2^, IQR: 2.80–4.85) than the tibialis anterior (4.80 kg/cm^2^, IQR: 3.77–8.30), indicating greater mechanical pain sensitivity at the trapezius site. BPI scores indicated moderate-to-severe clinical pain, with a median “worst pain” score of 7 (IQR: 7–8) and an “average pain” score of 5 (IQR: 4–7). Severe functional impact was highlighted by a median FIQ total score of 63.6 (IQR: 55.8–79.5) and a median CSI score of 66 (IQR: 60–74).

Lastly, median circulating concentrations of IL-6, TNF-α, and IL-10 were 2.20 pg/mL (IQR: 1.60–3.75, n = 20), 49.70 pg/mL (IQR: 24.05–70.75, n = 19), and 14.80 pg/mL (IQR: 11.20–20.00, n = 13), respectively.

### 3.2. Exploratory Correlation Analysis

#### 3.2.1. Association Between Lifestyle Factors and Pain Related Outcomes

**Figure 3:**
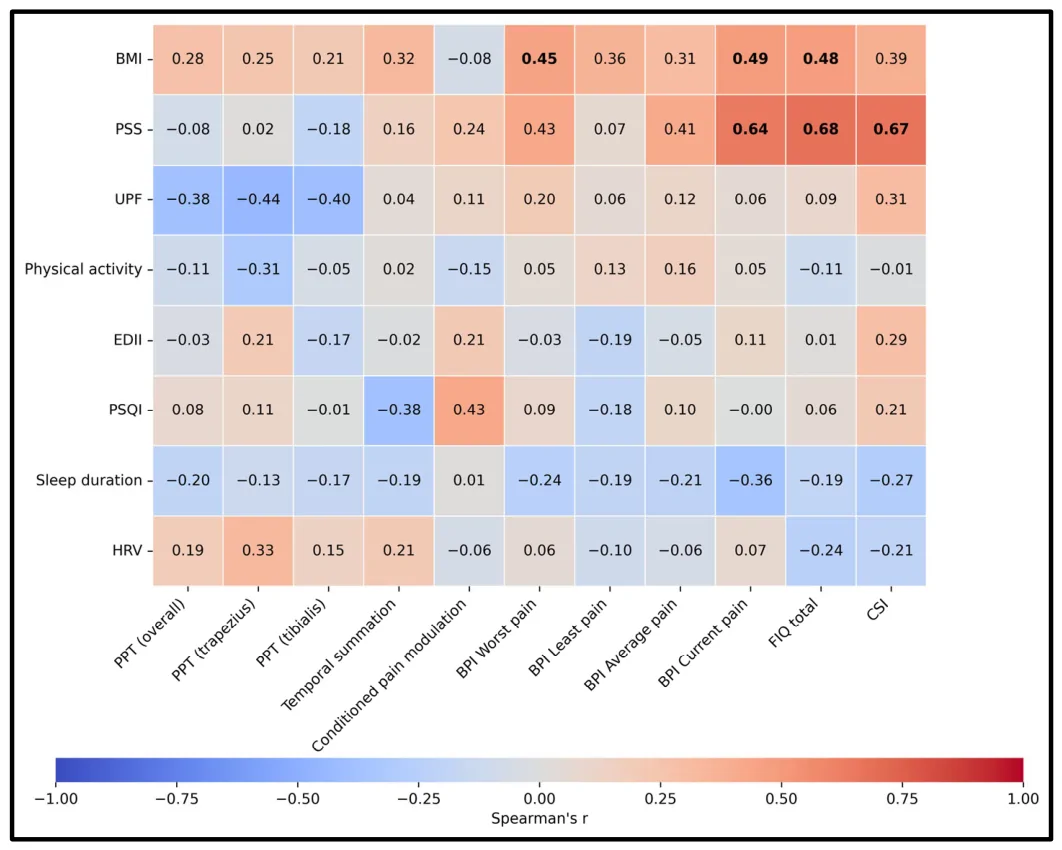
Clustered Spearman correlation heatmap illustrating relationships between lifestyle factors and pain outcomes in women with fibromyalgia. Bold values indicate statistical significance (*p* < 0.05). Spearman’s rank correlation coefficients (r) are displayed within each cell. Positive correlations are shown in shades of red and negative correlations in shades of blue, with colour intensity reflecting the strength of the association. Hierarchical clustering was applied to group variables with similar correlation patterns. Abbreviations: EDII, Energy-adjusted Dietary Inflammatory Index; UPF, ultra-processed food; BMI, body mass index; HRV, heart rate variability; PSQI, Pittsburgh Sleep Quality Index; PSS, Perceived Stress Scale; PPT, pressure pain threshold; BPI, Brief Pain Inventory; FIQ, Fibromyalgia Impact Questionnaire; CSI, Central Sensitisation Inventory.

Associations between lifestyle factors and pain-related outcomes are illustrated in the clustered Spearman correlation heatmap (Figure 3), with the corresponding correlation coefficients, 95% confidence intervals, and *p*-values presented in Supplementary Table S3.

Perceived stress measured with PSS demonstrated the strongest associations with pain-related outcomes. Higher PSS scores were strongly correlated with greater fibromyalgia impact (r = 0.68, 95% CI: 0.24–0.93, *p* < 0.001), higher CSI scores (r = 0.67, 95% CI: 0.31–0.81 *p* < 0.001), and greater current BPI pain intensity (r = 0.64, 95% CI: 0.21–0.81, *p* = 0.002). Moderate positive associations were also observed between PSS and worst pain (r = 0.43, 95% CI: 0–0.74, *p* = 0.059) and average pain (r = 0.41, 95% CI: −0.07–0.76, *p* = 0.072).

BMI showed moderate positive correlations with current BPI pain intensity (r = 0.49, 95% CI: 0.13–0.7, *p* = 0.028), fibromyalgia impact (r = 0.48, 95% CI: 0.13–0.72, *p* = 0.031), and worst BPI pain intensity (r = 0.45, 95% CI: 0.07–0.73, *p* = 0.049). A weak-to-moderate positive association was also observed between BMI and CSI scores (r = 0.39, 95% CI: −0.05–0.71, *p* = 0.089).

Regarding dietary factors, greater consumption of UPF was moderately associated with lower pressure pain thresholds at both the trapezius (r = −0.44, 95% CI: −0.81–0.06, *p* = 0.054) and tibialis anterior (r = −0.40, 95% CI: −0.73–0.05, *p* = 0.080), with the negative direction indicating lower pressure pain threshold with greater UPF intake.

Regarding sleep-related measures, poorer subjective sleep quality (higher PSQI score) showed a moderate positive correlation with CPM (r = 0.43, 95% CI: −0.09–0.82, *p* = 0.060), while a weak -to-moderate negative correlation was observed between PSQI score and TS (r = −0.38, 95% CI: −0.77–0.13, *p* = 0.101). Fitbit-derived sleep duration, daily physical activity, and HRV showed weak insignificant associations with the assessed pain-related outcomes.

#### 3.2.2. Association Between Lifestyle Factors and Inflammatory Biomarkers

**Figure 4:**
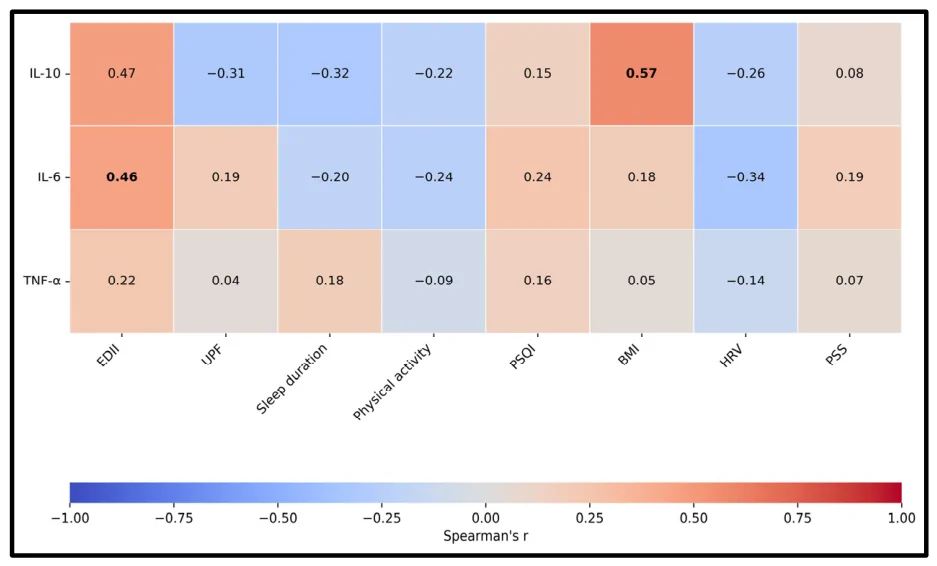
Clustered Spearman correlation heatmap illustrating relationships between lifestyle factors and inflammatory biomarkers in women with fibromyalgia. Bold values indicate statistical significance (*p* < 0.05). Note: Quantifiable cytokine concentrations were available for all 20 participants for IL-6, 13 participants for IL-10, and 19 participants for TNF-α because some measurements were below the quantitative range of the respective assays. Spearman’s rank correlation coefficients (r) are displayed within each cell. Positive correlations are shown in shades of red and negative correlations in shades of blue, with colour intensity reflecting the strength of the association. Hierarchical clustering was applied to group variables with similar correlation patterns. Abbreviations: EDII, Energy-adjusted Dietary Inflammatory Index; UPF, ultra-processed food; BMI, body mass index; HRV, heart rate variability; PSQI, Pittsburgh Sleep Quality Index; PSS, Perceived Stress Scale; IL-6, interleukin-6; TNF-α, tumour necrosis factor-alpha; IL-10, interleukin-10.

Associations between lifestyle factors and inflammatory biomarkers are presented in the clustered Spearman correlation heatmap (Figure 4), with the corresponding correlation coefficients, 95% confidence intervals, and *p*-values reported in Supplementary Table S4.

The EDII demonstrated moderate positive associations with inflammatory biomarkers. A more pro-inflammatory diet showed moderate-to-strong positive correlations with circulating concentrations of IL-6 (r = 0.46, 95% CI: −0.02–0.79, *p* = 0.04, n = 20) and IL-10 (r = 0.47, 95% CI: −0.16–0.87, *p* = 0.10, n = 13). In contrast, the association between EDII and TNF-α was weak and statistically insignificant (r = 0.22, 95% CI: −0.3–0.67, *p* = 0.36, n = 19).

BMI also showed a moderate-to-strong positive correlation with IL-10 concentrations (r = 0.57, 95% CI: −0.12–0.93, *p* = 0.042, n = 13), whereas the correlations with IL-6 (n = 20) and TNF-α (n = 19) were weaker in magnitude.

No moderate-to-strong associations were identified between inflammatory biomarkers and UPF consumption, Fitbit-derived physical activity, sleep duration, HRV, PSS, and PSQI.

#### 3.2.3. Association Between Inflammatory Biomarkers and Pain-Related Outcomes

**Figure 5:**
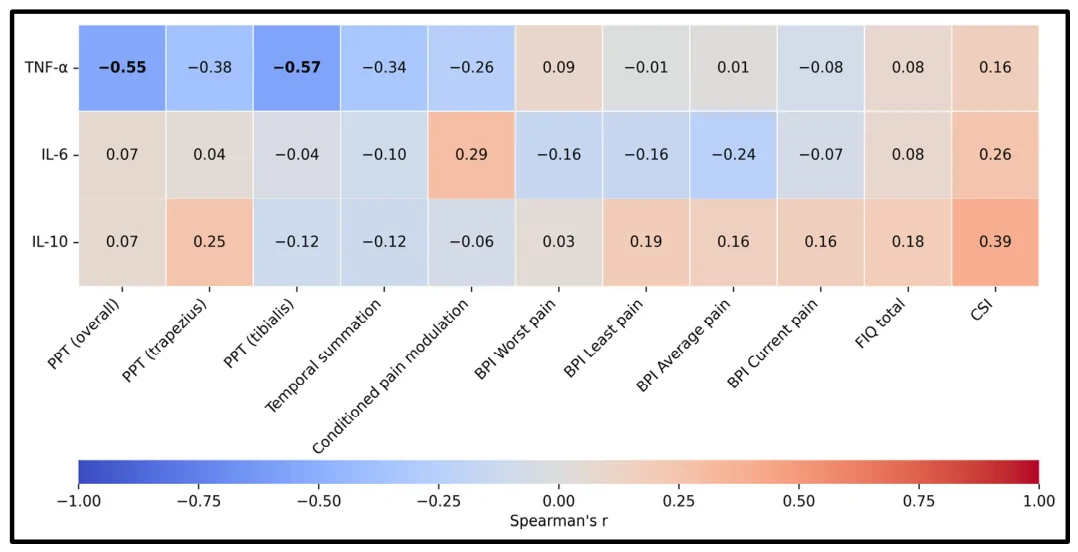
Clustered Spearman correlation heatmap illustrating relationships between inflammatory biomarkers and pain outcomes in women with fibromyalgia. Bold values indicate statistical significance (*p* < 0.05). Note: Quantifiable cytokine concentrations were available for all 20 participants for IL-6, 13 participants for IL-10, and 19 participants for TNF-α because some measurements were below the quantitative range of the respective assays. Spearman’s rank correlation coefficients (r) are displayed within each cell. Positive correlations are shown in shades of red and negative correlations in shades of blue, with colour intensity reflecting the strength of the association. Hierarchical clustering was applied to group variables with similar correlation patterns. Abbreviations: IL-6, interleukin-6; TNF-α, tumour necrosis factor-alpha; IL-10, interleukin-10; PPT, pressure pain threshold; BPI, Brief Pain Inventory; FIQ, Fibromyalgia Impact Questionnaire; CSI, Central Sensitisation Inventory.

Associations between inflammatory biomarkers and pain-related outcomes are illustrated in the clustered Spearman correlation heatmap (Figure 5), with the corresponding correlation coefficients, 95% confidence intervals, and *p*-values presented in Supplementary Table S5.

TNF-α demonstrated the strongest associations with pain-related outcomes. Higher TNF-α concentrations were moderately negatively correlated with lower overall PPT (r = −0.55, 95% CI: −0.79–−0.16, *p* = 0.021, n = 19) and lower tibialis anterior PPT (r = −0.57, 95% CI: −0.81–−0.19, *p* = 0.010, n = 19). A weak negative association was also observed between TNF-α and trapezius PPT (r = −0.38, 95% CI: −0.72–0.1, *p* = 0.106, n = 19).

IL-6 (n = 20) showed no moderate-to-strong associations with the assessed pain-related outcomes. Similarly, IL-10 (n = 13) showed predominantly weak associations with pain sensitivity, pain modulation, pain intensity, FIQ, and CSI. A weak-to-moderate positive association was observed between IL-10 and CSI (r = 0.39, 95% CI: −0.20–0.78, n = 13).

### 3.3. Exploratory Adjusted Regression Analysis

Exploratory multiple linear regression analyses were performed for the moderate-to-strong associations (|r| ≥ 0.40) identified in the correlation analyses and adjusted for age and disease duration. Regression analyses for smoking status and alcohol intake were not performed because these variables were analysed as ordinal categorical variables. The regression results are presented in Table 2.

In the adjusted regression analyses, higher PSS scores were associated with greater fibromyalgia impact (β = 2.27, 95% CI: 1.45–3.09), higher CSI scores (β = 1.69, 95% CI: 1.07–2.31), greater current BPI pain intensity (β = 0.30, 95% CI: 0.17–0.44), and worst BPI pain intensity (β = 0.14, 95% CI: 0.03–0.25). Higher EDII scores were also associated with higher IL-6 (β = 1.39, 95% CI: 0.86–1.90) and IL-10 concentrations (β = 0.05, 95% CI: 0.005–0.088). In addition, higher BMI was associated with higher IL-10 concentrations (β = 1.42, 95% CI: 0.85–1.99). The remaining regression models showed smaller effect estimates, with confidence intervals that included zero.

### 3.4. False Discovery Rate Sensitivity Analysis

To evaluate the robustness of the primary findings against multiple testing, False Discovery Rate (FDR) corrections were applied using the Benjamini–Hochberg procedure. Following FDR adjustment, none of the associations identified in the unadjusted analyses met the predefined FDR threshold. The complete unadjusted and FDR-adjusted results for all analyses are presented in Supplementary Tables S3–S5.

## 4. Discussion

### 4.1. Summary of the Main Findings

This exploratory cross-sectional pilot study investigated the relationships between multimodal modifiable lifestyle factors, systemic inflammatory biomarkers, and pain-related objective and subjective outcomes in women with fibromyalgia. Overall, perceived stress demonstrated the strongest and most consistent associations with greater pain intensity, central sensitization symptoms, and fibromyalgia impact, with similar effect directions and magnitudes observed after adjustment for age and disease duration. A more pro-inflammatory diet as measured using EDII showed moderate positive associations with higher circulating concentrations of IL-6 (n = 20) and IL-10 (n = 13), while higher BMI showed a moderate positive association IL-10 concentrations. In contrast, objectively measured physical activity, sleep duration, and heart rate variability showed a weak insignificant association with pain-related outcomes or inflammatory biomarkers in this exploratory small cohort. Ultra-processed food intake showed moderate negative associations with selected pressure pain threshold measures, whereas TNF-α (n = 19) was the only inflammatory biomarker showing moderate associations with pressure pain thresholds. The FDR sensitivity analysis did not retain any of the associations identified using the unadjusted *p*-value threshold, which emphasizes that these findings should be interpreted as preliminary effect-size estimates rather than confirmatory evidence.

### 4.2. Perceived Stress, Pain Processing, and Inflammation

The stronger and more consistent associations involving perceived stress distinguish it from the other lifestyle factors examined in this study. In the regression model adjusted for age and ‘time since diagnosis’, each one-unit increase in PSS score was associated with a 2.27-point higher FIQ score, a 1.69-point higher CSI score, and higher current and worst pain intensity. Although the magnitude and direction of these associations were maintained after adjustment for age and disease duration, other potentially relevant covariates, such as depression, anxiety, catastrophising, medication use, and sleep quality, were not included in the regression models to minimise overfitting given the small sample size and pilot nature of this study. Residual confounding by these unmeasured factors therefore cannot be excluded.

Stress has long been implicated in pain amplification across chronic pain conditions more broadly, typically through dysregulation of descending pain modulation and hypothalamic–pituitary–adrenal (HPA) axis function [33]. However, this relationship is particularly evident in fibromyalgia, which is increasingly recognised as a stress-associated disorder [34]. In fibromyalgia, perceived psychological stress may have a stronger relationship with clinical severity than biological stress measures such as cortisol [35]. Supporting this, a recent study comparing individuals with fibromyalgia and pain-free controls found that perceived stress, but not salivary or hair cortisol, was associated with fibromyalgia status, symptom burden, pain severity, and pain interference. The mean PSS score in the fibromyalgia group was approximately 21 [35]. In our study, the median PSS score was 25 (IQR 20–28), alongside a median FIQ score of 63.6 and CSI score of 66, indicating substantial disease impact, central sensitisation symptoms, and perceived stress in the study sample. This pattern is consistent with evidence suggesting that perceived psychological stress, rather than endocrine stress responses, is more closely linked to central pain processing in fibromyalgia [36]. Mechanistically, chronic psychological stress may sensitise pain pathways through activation of the sympathetic nervous system and HPA axis, glial and microglial activation, and release of pro-inflammatory mediators within the central nervous system, alongside impaired descending pain inhibition, collectively lowering the threshold for pain amplification and central sensitisation [33].

Notably, in this study, PSS was not associated with any of the inflammatory biomarkers assessed. This may indicate that the relationship between perceived stress and pain-related outcomes is not necessarily mediated through the peripheral inflammatory markers measured in this study. Alternatively, the small sample size and single time-point assessment of circulating biomarkers may have limited the ability to detect relationships between perceived stress and peripheral inflammation.

Sex- and hormone-related factors may also contribute to this relationship. Fibromyalgia predominantly affects women, and ovarian hormones may influence both nociceptive processing and stress-related physiological responses [37]. Changes in oestrogen and progesterone across the menstrual cycle, as well as the hormonal changes accompanying the menopausal transition, have been linked to alterations in pain sensitivity and may contribute to variability in fibromyalgia symptoms across the female lifespan [37,38]. Consequently, variation in menopausal status, menstrual-cycle phase, or exogenous hormone use, none of which were assessed in the present study, may have contributed to inter-individual differences in pain and perceived stress.

### 4.3. Dietary Factors, Pain Processing, and Inflammation

Regarding dietary factors, a more pro-inflammatory diet, as measured using EDII, showed a moderate positive association with central sensitisation symptoms in the Spearman correlation analysis, although the confidence interval included zero and the association did not remain following adjustment for age and disease duration. This pattern suggests that the relationship between diet and central pain processing may be less consistent than the relationship observed between diet and circulating inflammatory markers.

Chronic pain is increasingly recognised as having an inflammatory component, with low-grade systemic inflammation contributing to peripheral and central sensitisation through cytokine-mediated sensitisation of nociceptors and glial activation [39]. Pro-inflammatory dietary patterns, characterised by high intake of refined carbohydrates and saturated fats and low intake of fibre, antioxidants, and omega-3 fatty acids, have been proposed to promote systemic inflammation via activation of pro-inflammatory signalling pathways and alterations in gut microbial composition and intestinal permeability [40]. Higher DII scores have previously been associated with greater chronic pain prevalence in population-based studies [13,41] and, more specifically, with pain hypersensitivity and pressure pain thresholds in women with fibromyalgia [11,42], as well as with higher pain, disease severity, and inflammatory biochemical parameters [43].

In the present study, EDII showed moderate-to-strong positive correlations with both IL-6 and IL-10, and the direction and magnitude of these associations were broadly maintained in the adjusted regression models. This consistency across both the correlation and regression analyses is broadly in line with this literature and provides a basis for further investigation of diet–inflammation relationships in fibromyalgia [41,42,43]. However, these findings should be interpreted cautiously because none of the associations retained the FDR-adjusted threshold in the sensitivity analysis.

The positive association between E-DII and IL-10 needs particular consideration as this analysis was based on only 13 participants with quantifiable IL-10 concentrations. Although IL-10 is generally regarded as an anti-inflammatory cytokine, its elevation in the context of a more pro-inflammatory diet may reflect a compensatory regulatory response to increased inflammatory signalling, rather than simply indicating an anti-inflammatory physiological state [44]. A similar pattern was observed in this study for BMI, which showed a positive association with IL-10 concentrations but not with IL-6 or TNF-α. This is consistent with the possibility that adiposity contributes to systemic immune activation through metabolic and immune pathways, with IL-10 upregulation potentially representing a compensatory attempt to counterbalance adiposity-related pro-inflammatory signalling; inflamed adipose tissue is itself known to secrete both pro- and anti-inflammatory mediators, including IL-10, alongside classical pro-inflammatory cytokines [45]. However, BMI is a relatively crude measure of adiposity that does not distinguish fat mass, lean mass, or visceral adiposity, and future studies should consider more detailed body composition measures when examining this relationship.

In contrast, associations between ultra-processed food consumption and pain-related measures were limited, and no moderate-to-strong associations were observed between UPF intake and the inflammatory biomarkers assessed. This is not unusual; a recent scoping review of the relationship between UPF consumption and systemic inflammatory biomarkers in adults reported inconsistent findings across cytokines, including an absence of association with IL-1β and mixed findings for IL-6 and TNF-α, and highlighted substantial methodological heterogeneity in how UPF intake and inflammation have been assessed across studies [46]. This contrast may also partly reflect a methodological difference between the two dietary measures used in this study: the EDII was itself constructed and weighted using literature-derived relationships between individual dietary components and circulating inflammatory biomarkers, including IL-6, IL-10, and TNF-α, whereas UPF classification is based on the degree of food processing rather than on an established link to specific inflammatory pathways [24]. It is therefore plausible that the EDII was inherently better placed to capture associations with the inflammatory biomarkers measured here than UPF intake, which indexes a related but conceptually distinct dietary construct. This suggests that the null findings for UPF observed here may reflect genuine complexity in the relationship between food processing, nutrient density, and inflammatory or pain outcomes, rather than a true absence of effect, and may also reflect limitations inherent to UPF intake measurement more broadly.

### 4.4. Sleep, Physical Activity, HRV, Pain Processing, and Inflammation

The objectively measured physical activity, sleep duration, and HRV measures showed no moderate-to-strong associations with the assessed pain-related outcomes or inflammatory biomarkers. This contrasts with the stronger patterns observed for subjective measures such as perceived stress and, in some analyses, subjective sleep quality. Several explanations might be possible here. First, with a sample of 20 participants, statistical power to detect modest associations was limited, and true effects may have gone undetected. Second, the cohort had relatively limited variability in subjective sleep quality, with a median PSQI score of 12 (IQR 10–13), which may have restricted the ability to detect associations even where they exist. Sleep is also multidimensional, and sleep duration alone may not adequately capture fragmentation, circadian timing, sleep architecture, or insomnia symptoms relevant to pain [47].

An unexpected finding was the moderate positive association between PSQI score and CPM (r = 0.43, 95% CI: −0.09–0.82). Higher PSQI scores represent poorer subjective sleep quality, whereas higher CPM values in this protocol reflect greater change in pressure pain threshold following the conditioning cuff stimulus indicating greater endogenous pain inhibitory capacity. Thus, poorer subjective sleep quality was associated with greater CPM responses in this sample, rather than with reduced pain inhibition as might have been anticipated. This finding should be interpreted cautiously considering the exploratory design and the imprecision of the confidence interval. It may reflect the small sample size, variability in individual pain-modulation responses, or the distinction between subjective sleep quality and experimentally assessed pain modulation.

Sleep disturbance is commonly reported in fibromyalgia and has been linked to pain and inflammatory processes; however, the magnitude of relationships between sleep and systemic inflammation appears modest. A systematic review and meta-analysis of 72 studies found that sleep disturbance was associated with small increases in CRP and IL-6, whereas sleep duration showed more limited and inconsistent relationships, and neither sleep disturbance nor sleep duration was associated with TNF-α [48]. More recent work using network analysis in 1536 people with chronic musculoskeletal pain found that direct relationships between subjective sleep and pain variables were very weak, challenging the assumption that sleep and pain are necessarily tightly coupled at the individual level [49]. Third, Fitbit-derived step counts, sleep duration, and HRV, while practical, do not represent gold-standard physiological measurements, particularly for HRV, and may have introduced measurement error that attenuated true associations [50]. It is notable that, in contrast to these objective measures, subjective indicators such as perceived stress and PSQI score did show associations with pain-related outcomes, raising the possibility that the contrast reflects, at least in part, a distinction between subjective appraisal and objective behavioural quantification rather than a true absence of a physiological relationship.

### 4.5. Inflammation and Pain Processing

Among the inflammatory biomarkers assessed, TNF-α (n = 19) showed the strongest associations with pain-related outcomes, with higher concentrations associated with lower overall and tibialis anterior pressure pain thresholds, indicating greater mechanical pain sensitivity. Notably, TNF-α showed weaker associations with subjective pain intensity, FIQ, central sensitization symptoms, temporal summation, or conditioned pain modulation, suggesting that systemic TNF-α concentrations may be more closely linked to specific aspects of peripheral and quantitative sensory processing than to the broader clinical experience of fibromyalgia [51].

This pattern is consistent with mechanistic evidence implicating TNF-α in nociceptor sensitisation and neuroimmune activation; experimental work using human blood-derived microglia-like cells has demonstrated hyperresponsive TNF-α production in fibromyalgia patients relative to healthy individuals, with correlations between TNF-α upregulation and clinical pain parameters [33], while broader reviews of neuropathic pain mechanisms describe a pivotal role for TNF-α in peripheral and central sensitisation via actions at the site of injury, dorsal root ganglion, spinal dorsal horn, and higher pain-processing centres [51]. A recent systematic review and meta-analysis of circulating cytokines in fibromyalgia similarly reported consistently elevated TNF-α, IL-6, and IL-8 alongside elevated IL-10 in fibromyalgia patients compared with healthy controls, supporting the existence of an altered, if complex, peripheral cytokine profile in this population [52].

Although IL-6 (n = 20) and IL-10 (n = 13) were also associated with dietary and adiposity measures, the weaker patterns between these cytokines and pain-related outcomes, contrasted with the pattern observed for TNF-α, raise the possibility that different inflammatory pathways may relate to different components of the fibromyalgia phenotype. However, the present exploratory data are insufficient to establish whether TNF-α is more proximally involved in pain generation or maintenance than IL-6 or IL-10.

### 4.6. Strengths and Limitations

This study has several strengths. A key strength of this exploratory study is the integration of multimodal assessments of lifestyle behaviours, circulating inflammatory biomarkers, quantitative sensory testing, wearable-derived physiological measures, and patient-reported outcomes in women with fibromyalgia. Lifestyle assessment incorporated both subjective and objective measures, including Fitbit-derived physical activity, sleep duration, and heart rate variability, alongside detailed three-day dietary records used to calculate the EDII and ultra-processed food intake. In addition, pain was characterised using validated questionnaires together with quantitative sensory testing, providing a comprehensive assessment of both clinical symptoms and pain processing mechanisms. All recruited participants completed the study, with no dropouts and complete datasets available for analysis Although exploratory, this interdisciplinary study provides preliminary evidence on the relationships between multiple lifestyle, physiological, biological, and clinical domains in fibromyalgia, generating effect size estimates and hypotheses that may inform the design of future longitudinal and interventional studies.

On the other hand, several limitations should be considered when interpreting the findings of this study. First, the small sample size (n = 20) limits statistical power and increases the risk of both type I and type II errors; therefore, the results should be considered exploratory. Second, the cross-sectional observational design prevents us establishing causal relationships or determining the direction of the observed associations. Third, only women with fibromyalgia were included, limiting the generalisability of the findings to men and to individuals with other chronic pain conditions.

The sample sizes for analyses involving IL-10 and TNF-α were reduced because seven IL-10 and one TNF-α measurements were below the quantitative range of the respective assays, resulting in analytical sample sizes of n = 13 and n = 19, respectively. This may have reduced statistical power, particularly for the IL-10 analyses.

Although wearable devices provide objective estimates of physical activity, sleep, and HRV, they do not represent gold-standard physiological measurements. In particular, ECG is considered the gold-standard method for HRV assessment [53], whereas polysomnography (PSG) is the gold-standard method for objective sleep assessment [54].

Finally, the regression analyses were intentionally restricted to a small number of prespecified covariates (age and time since fibromyalgia diagnosis) to minimise model overfitting. As a result, other potentially important confounding factors were not included. For example, BMI was not included as a covariate, and therefore potential confounding by adiposity cannot be excluded. These regression analyses were intended to explore associations and identify potential relationships rather than provide causal estimates.

### 4.7. Future Recommendations

Larger, adequately powered longitudinal studies using appropriate causal inference methods are needed to confirm these associations, establish temporality, and provide stronger evidence regarding potential causal relationships. Importantly, this study provides preliminary effect size estimates that may help inform sample size calculations and the design of future larger trials. Future research, using appropriate causal inference methodology, should investigate whether modifications in multimodal modifiable lifestyle factors, including diet, stress, sleep, physical activity, smoking, alcohol consumption, and body composition, can lead to improvements in inflammatory biomarker profiles, pain processing, and clinical pain outcomes in people with fibromyalgia.

Randomised controlled trials evaluating multidisciplinary multimodal lifestyle interventions that target these modifiable lifestyle factors may provide important evidence for more personalised fibromyalgia management including the use of remote monitoring devices to support real-life, real-time data collection alongside gold-standard physiological assessments, such as polysomnography for sleep and electrocardiography for heart rate variability, where feasible. In addition, integrating a broader range of inflammatory and metabolic biomarkers, together with advanced metabolomics and other multi-omics techniques, may further elucidate the biological pathways linking modifiable lifestyle behaviours with chronic pain and identify potential therapeutic targets.

## 5. Conclusions

This exploratory pilot study provides novel insights into the complex relationships between multimodal modifiable lifestyle factors, systemic inflammatory biomarkers, and pain-related outcomes in women with fibromyalgia. Perceived stress demonstrated the strongest and most consistent associations with pain intensity, central sensitization symptoms, and fibromyalgia impact, while a more pro-inflammatory diet and higher BMI showed moderate associations with selected inflammatory biomarkers. Higher TNF-α concentrations were also associated with greater mechanical pain sensitivity, while ultra-processed food intake showed moderate associations with selected pressure pain sensitivity measures. In contrast, objectively measured physical activity, sleep duration, and heart rate variability showed no moderate-to-strong associations with the assessed outcomes in this exploratory cohort.

Although the findings should be interpreted cautiously because of the exploratory cross-sectional design, small sample size, and multiple comparisons, they provide preliminary effect-size estimates that may help inform future hypothesis-driven research. The observed patterns suggest that stress, dietary factors, adiposity, inflammatory processes, and pain sensitivity may represent interconnected components of the broader biological and behavioural context of fibromyalgia, rather than isolated pathways. However, none of the observed associations remained after false discovery rate correction, and therefore the findings should be considered hypothesis-generating rather than confirmatory.

By integrating nutritional, behavioural, physiological, inflammatory, and pain-related assessments within a single interdisciplinary framework, this study provides a useful proof of concept for multimodal lifestyle medicine research in chronic pain and demonstrates the feasibility of this approach for larger studies. Larger, adequately powered longitudinal and interventional studies are now warranted to determine whether these relationships are reproducible, clarify potential causal pathways, and establish whether targeting multiple modifiable lifestyle factors simultaneously can improve inflammation, pain processing, and clinical outcomes in people with fibromyalgia.

## Figures and Tables

**Table 1:** Demographic and Baseline Characteristics.

Variable	Value
Age (years), Median (IQR)	48 (35–51)
Sex, Female, n (n%)	20 (100%)
Months since diagnosis (months), Median (IQR)	66 (18–90)
BMI (kg/m^2^), Median (IQR)	28.1 (22.9–39.6)
EDII (score), Median (IQR)	3.32 (1.28–4.03)
UPF (% energy), Median (IQR)	50.0 (37.8–60.6)
Smoking status, n (%)	
Current	2 (10.0%)
Never	10 (50.0%)
Previous	8 (40.0%)
Alcohol intake, n (%)	
Daily or almost daily	1 (5.0%)
Three or four times a week	0 (0%)
Once or twice a week	5 (25.0%)
One to three times a month	4 (20.0%)
Special occasions	4 (20.0%)
Previous Never	4 (20.0%) 2 (10.0%)
Fitbit physical activity (steps/day), Median (IQR)	6140 (4366–9343)
Fitbit sleep duration (hours/night), Median (IQR)	7.5 (6.7–7.7)
PSQI (score), Median (IQR)	12 (10–13)
PSS (score), Median (IQR)	25 (20–28)
HRV (ms), Median (IQR)	26.8 (20.9–35.0)
PPT overall average (kg/cm^2^), Median (IQR)	4.35 (3.27–6.70)
Average trapezius PPT (kg/cm^2^), Median (IQR)	4.05 (2.80–4.85)
Average tibialis PPT (kg/cm^2^), Median (IQR)	4.80 (3.77–8.30)
Temporal summation, (score), Median (IQR)	2 (2–3)
Conditioned pain modulation (kg/cm^2^), Median (IQR)	1.15 (0.30–2.23)
BPI worst pain (score), Median (IQR)	7 (7–8)
BPI least pain (score), Median (IQR)	3 (2–4)
BPI average pain (score), Median (IQR)	5 (4–7)
BPI current pain (score), Median (IQR)	6 (4–8)
FIQ total (score), Median (IQR)	63.6 (55.8–79.5)
CSI (score), Median (IQR)	66 (60–74)
IL-6 (pg/mL), Median (IQR)	2.20 (1.60–3.75)
TNF-α (pg/mL), Median (IQR)	49.70 (24.05–70.75)
IL-10 (pg/mL), Median (IQR)	14.80 (11.20–20.00)

Note: There were no participant dropouts, and data and plasma samples were available for all 20 participants. Quantifiable cytokine concentrations were available for all 20 participants for IL-6, 13 participants for IL-10, and 19 participants for TNF-α because some measurements were below the quantitative range of the respective assays. Abbreviations: IQR, interquartile range; n, number of participants; BMI, body mass index; PSQI, Pittsburgh Sleep Quality Index; PSS, Perceived Stress Scale; HRV, heart rate variability; EDII, Energy-adjusted Dietary Inflammatory Index; UPF, ultra-processed food; PPT, pressure pain threshold; BPI, Brief Pain Inventory; FIQ, Fibromyalgia Impact Questionnaire; CSI, Central Sensitisation Inventory; IL-6, interleukin-6; TNF-α, tumour necrosis factor-alpha; IL-10, interleukin-10; pg/mL, picograms per millilitre; kg/m^2^, kilograms per square metre; ms, milliseconds.

**Table 2:** Moderate-to-strong associations between lifestyle, inflammatory, and pain-related variables identified by Spearman’s correlation analyses, with corresponding exploratory age- and disease-duration-adjusted multiple linear regression results.

Predictor	Outcome	Spearman Rho	Direction	β (95% CI)	*p*	Adjusted R^2^
PSS	FIQ total	0.68	Positive	2.27 (1.45–3.09)	**0.001**	0.63
PSS	CSI	0.67	Positive	1.69 (1.07–2.31)	**0.001**	0.63
PSS	BPI current pain	0.64	Positive	0.30 (0.17–0.44)	**0.001**	0.51
BMI	IL-10	0.57	Positive	1.42 (0.85–1.99)	**0.001**	0.78
TNF-α	PPT tibialis	−0.57	Negative	−0.018 (−0.06–0.02)	0.37	−0.12
TNF-α	PPT overall	−0.55	Negative	−0.01 (−0.04–0.02)	0.34	−0.11
BMI	BPI current pain	0.49	Positive	0.04 (−0.04–0.13)	0.29	−0.10
BMI	FIQ total	0.48	Positive	0.44 (−0.13–1.00)	0.12	−0.01
EDII	IL-10	0.47	Positive	0.05 (0.005–0.088)	**0.03**	0.31
EDII	IL-6	0.46	Positive	1.39 (0.86–1.9)	**0.001**	0.64
BMI	BPI worst pain	0.45	Positive	0.03 (−0.03–0.08)	0.31	−0.09
UPF	PPT trapezius	−0.44	Negative	−0.09 (−0.19–0.01)	0.07	0.09
PSQI	CPM	0.43	Positive	0.30 (−0.10–0.70)	0.13	0.02
PSS	BPI worst pain	0.43	Positive	0.14 (0.03–0.25)	**0.02**	0.20
PSS	BPI average pain	0.41	Positive	0.13 (−0.00–0.26)	0.05	0.078
UPF	PPT tibialis	−0.40	Negative	−0.09 (−0.29–0.12)	0.37	−0.11

**Bold** indicates statistical significance which was set at *p* < 0.05. Note: Quantifiable cytokine concentrations were available for all 20 participants for IL-6, 13 participants for IL-10, and 19 participants for TNF-α because some measurements were below the quantitative range of the respective assays. Abbreviations: BMI, body mass index; BPI, Brief Pain Inventory; CI, confidence interval; CPM, conditioned pain modulation; CSI, Central Sensitization Inventory; EDII, Energy-adjusted Dietary Inflammatory Index; FIQ, Fibromyalgia Impact Questionnaire; IL-6, interleukin-6; IL-10, interleukin-10; PSS, Perceived Stress Scale; PPT, pressure pain threshold; PSQI, Pittsburgh Sleep Quality Index; TNF-α, tumour necrosis factor-alpha; UPF, ultra-processed food intake; β, unstandardised regression coefficient; r, Spearman’s rank correlation coefficient; Adjusted R^2^, adjusted coefficient of determination.

## Data Availability

The data that support the findings are available upon request from the corresponding author. The data are not publicly available due to privacy or ethical restrictions.

## Supplementary Materials

The following supporting information can be downloaded at: https://www.mdpi.com/article/10.3390/nu18213507/s1, Supplementary Figure S1: Quantile–quantile (Q–Q) plots for assessment of normality of continuous study variables. Each panel presents a Q–Q plot comparing the observed distribution of a continuous study variable with the theoretical normal distribution. Deviations from the reference line indicate departures from normality; Supplementary Table S1: NOVA food classification system used for the classification of ultra-processed foods, including category definition and representative examples; Supplementary Table S2: Results of the Shapiro–Wilk normality tests for continuous study variables; Supplementary Table S3: Bivariate associations between lifestyle factors and pain outcomes; Supplementary Table S4: Bivariate associations between lifestyle factors and inflammatory biomarkers; Supplementary Table S5: Bivariate associations between inflammatory biomarkers and pain outcomes.
