# Supplementary material for: Exploring Relationships Between Multimodal Lifestyle Factors, Inflammatory Biomarkers, and Pain Processing in Women with Fibromyalgia: An Exploratory Pilot Study
Source: Nutrients. 2026 Oct 25;18(21):3507. doi: 10.3390/nu18213507 (PMC13765189; doi:10.3390/nu18213507)
Supplement: Supplementary file 1 [file nutrients-18-03507-s001.zip › nutrients-4561403-supplementary.pdf]

**Supplementary Figure S1. Quantile–quantile (Q–Q) plots for assessment of normality of continuous study variables.** Each panel presents a Q–Q plot comparing the observed distribution of a continuous study variable with the theoretical normal distribution. Deviations from the reference line indicate departures from normality.

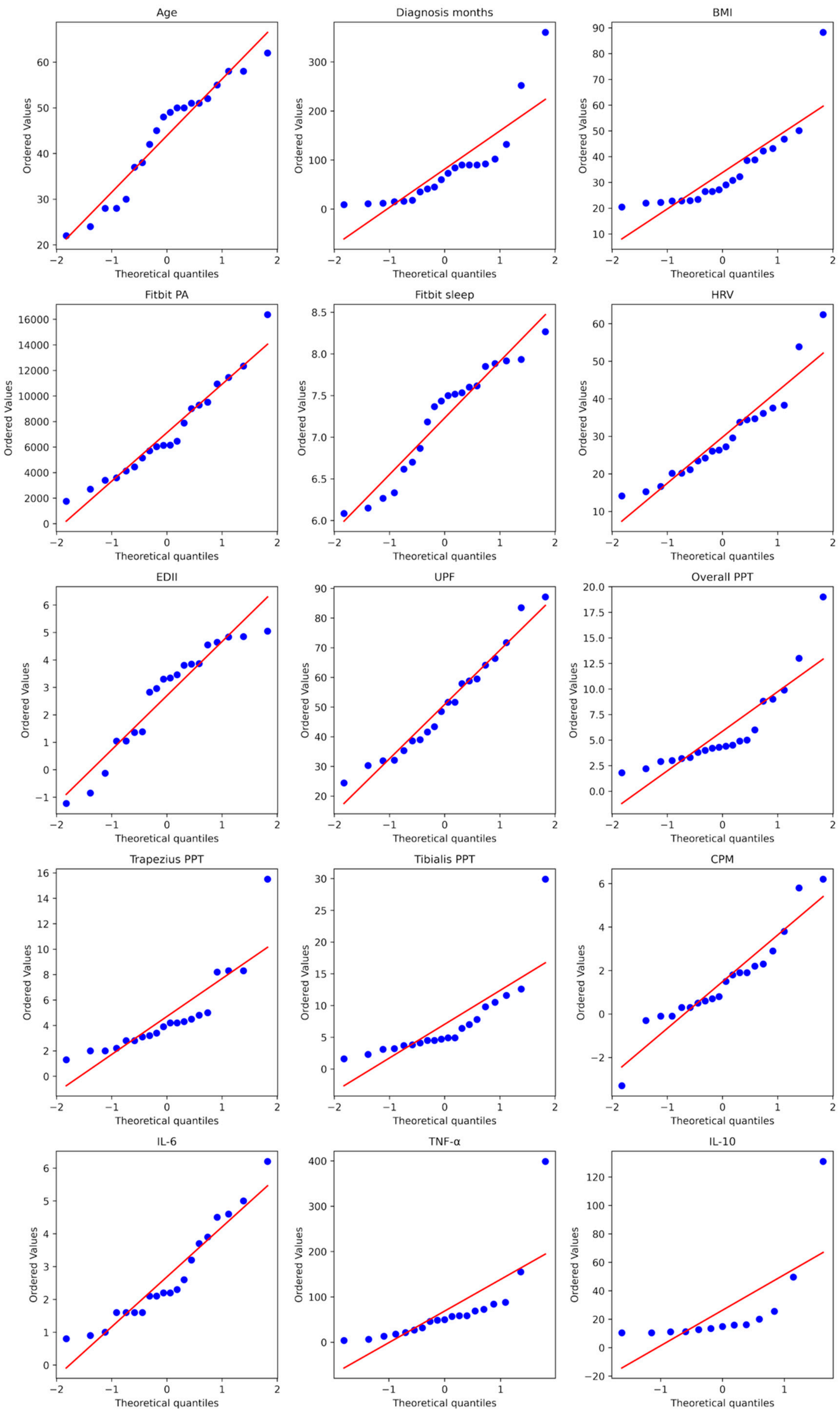
Age
Diagnosis months
BMI
Fitbit PA
Fitbit sleep
HRV
EDII
UPF
Overall PPT
Trapezius PPT
Tibialis PPT
CPM
IL-6
TNF-α
IL-10
Ordered Values
Theoretical quantiles

**Supplementary Table S1:** NOVA food classification system used for the classification of ultra-processed foods, including category definition and representative examples

| Degree of Processing | Definition | Examples |
|---|---|---|
| Ultra-processed foods | Industrial formulations made mostly or entirely from substances derived from foods (oils, fats, starches, sugars, protein isolates) and additives. Typically contain colourings, flavourings, emulsifiers, sweeteners or other additives to imitate or enhance sensory qualities or shelf-life. Often ready-to eat, hyper-palatable, and aggressively marketed. | Soft drinks, energy drinks, sweetened fruit drinks; packaged snacks (crisps, cereal bars); confectionery; reconstituted meat products (e.g. chicken nuggets, hot dogs); mass-produced packaged bread and cakes; sweetened breakfast cereals; flavoured yoghurts; instant noodles; ready meals; powdered meal replacements. |

**Supplementary Table S2:** Results of the Shapiro–Wilk normality tests for continuous study variables

| Variables | Shapiro-Wilk W | p-value |
|---|---|---|
| Age | 0.93 | 0.129 |
| Months since diagnosis | 0.74 | <0.001 |
| BMI | 0.74 | <0.001 |
| Fitbit physical activity | 0.95 | 0.345 |
| Fitbit sleep duration | 0.92 | 0.102 |
| PSQI | 0.95 | 0.377 |
| PSS | 0.96 | 0.498 |
| HRV | 0.90 | 0.046 |
| EDII | 0.91 | 0.053 |
| UPF | 0.96 | 0.476 |
| PPT overall average | 0.77 | <0.001 |
| Average trapezius PPT | 0.77 | <0.001 |
| Average tibialis PPT | 0.68 | <0.001 |
| Temporal summation | 0.94 | 0.246 |
| Conditioned pain modulation | 0.93 | 0.141 |
| BPI worst pain | 0.89 | 0.026 |
| BPI least pain | 0.95 | 0.400 |
| BPI average pain | 0.91 | 0.051 |
| BPI current pain | 0.92 | 0.111 |
| FIQ total | 0.95 | 0.366 |
| CSI | 0.96 | 0.608 |
| IL-6 | 0.92 | 0.078 |
| TNF-α | 0.60 | <0.001 |
| IL-10 | 0.52 | <0.001 |

**Note:** There were no participant dropouts, and data and plasma samples were available for all 20 participants. However, quantifiable cytokine concentrations were available for all 20 participants for IL-6, 13 participants for IL-10, and 19 participants for TNF-α because some measurements were below the quantitative range of the respective assays.

**Abbreviations:** BMI, Body Mass Index; PSQI, Pittsburgh Sleep Quality Index; PSS, Perceived Stress Scale; HRV, Heart Rate Variability; EDII, Empirical Dietary Inflammatory Index; UPF, Ultra-Processed Food; PPT, Pressure Pain Threshold; BPI, Brief Pain Inventory; FIQ, Fibromyalgia Impact Questionnaire; CSI, Central Sensitization Inventory; IL-6, Interleukin-6; TNF-α, Tumor Necrosis Factor-alpha; IL-10, Interleukin-10.

**Supplementary Table S3:** Bivariate associations between lifestyle factors and pain outcomes

| Variable 1 | Variable 2 | Spearman rho | 95% CI lower | 95% CI upper | p value | FDR p-value |
|---|---|---|---|---|---|---|
| EDII | PPT_average | -0.03 | -0.5 | 0.39 | 0.88 | 0.98 |
| EDII | average_trap | 0.21 | -0.33 | 0.65 | 0.38 | 0.89 |
| EDII | average_tib | -0.17 | -0.6 | 0.3 | 0.48 | 0.92 |
| EDII | TS_final | -0.02 | -0.47 | 0.48 | 0.95 | 0.99 |
| EDII | CPM_final | 0.21 | -0.26 | 0.6 | 0.36 | 0.89 |
| EDII | BPI_worst | -0.03 | -0.48 | 0.42 | 0.89 | 0.98 |
| EDII | BPI_least | -0.19 | -0.57 | 0.24 | 0.41 | 0.89 |
| EDII | BPI_average | -0.05 | -0.47 | 0.42 | 0.83 | 0.97 |
| EDII | BPI_current | 0.11 | -0.33 | 0.49 | 0.65 | 0.97 |
| EDII | FIQ_total | 0.01 | -0.47 | 0.48 | 0.97 | 0.99 |
| EDII | CSI | 0.29 | -0.17 | 0.67 | 0.22 | 0.88 |
| UPF | PPT_average | -0.38 | -0.75 | 0.11 | 0.10 | 0.64 |
| UPF | average_trap | -0.44 | -0.81 | 0.06 | 0.05 | 0.60 |
| UPF | average_tib | -0.40 | -0.73 | 0.05 | 0.08 | 0.64 |
| UPF | TS_final | 0.04 | -0.39 | 0.42 | 0.88 | 0.98 |
| UPF | CPM_final | 0.11 | -0.44 | 0.62 | 0.64 | 0.97 |
| UPF | BPI_worst | 0.20 | -0.39 | 0.68 | 0.40 | 0.89 |
| UPF | BPI_least | 0.06 | -0.44 | 0.55 | 0.80 | 0.97 |
| UPF | BPI_average | 0.12 | -0.43 | 0.6 | 0.62 | 0.97 |
| UPF | BPI_current | 0.06 | -0.51 | 0.56 | 0.80 | 0.97 |
| UPF | FIQ_total | 0.09 | -0.43 | 0.59 | 0.71 | 0.97 |
| UPF | CSI | 0.31 | -0.23 | 0.73 | 0.18 | 0.77 |
| fitbit_sleep | PPT_average | -0.20 | -0.56 | 0.24 | 0.41 | 0.89 |
| fitbit_sleep | average_trap | -0.13 | -0.51 | 0.3 | 0.60 | 0.97 |
| fitbit_sleep | average_tib | -0.17 | -0.57 | 0.29 | 0.48 | 0.92 |
| fitbit_sleep | TS_final | -0.19 | -0.56 | 0.21 | 0.41 | 0.89 |
| fitbit_sleep | CPM_final | 0.01 | -0.4 | 0.46 | 0.96 | 0.99 |
| fitbit_sleep | BPI_worst | -0.24 | -0.67 | 0.25 | 0.30 | 0.89 |
| fitbit_sleep | BPI_least | -0.19 | -0.62 | 0.32 | 0.43 | 0.89 |
| fitbit_sleep | BPI_average | -0.21 | -0.68 | 0.29 | 0.37 | 0.89 |
| fitbit_sleep | BPI_current | -0.36 | -0.79 | 0.15 | 0.12 | 0.66 |
| fitbit_sleep | FIQ_total | -0.19 | -0.63 | 0.31 | 0.43 | 0.89 |
| fitbit_sleep | CSI | -0.27 | -0.67 | 0.23 | 0.24 | 0.88 |
| fitbit_pa | PPT_average | -0.11 | -0.52 | 0.34 | 0.65 | 0.97 |
| fitbit_pa | average_trap | -0.31 | -0.66 | 0.13 | 0.18 | 0.77 |
| fitbit_pa | average_tib | -0.05 | -0.5 | 0.43 | 0.82 | 0.97 |
| fitbit_pa | TS_final | 0.02 | -0.45 | 0.48 | 0.94 | 0.99 |
| fitbit_pa | CPM_final | -0.15 | -0.55 | 0.32 | 0.54 | 0.95 |
| fitbit_pa | BPI_worst | 0.05 | -0.43 | 0.54 | 0.85 | 0.97 |
| fitbit_pa | BPI_least | 0.13 | -0.35 | 0.58 | 0.60 | 0.97 |
| fitbit_pa | BPI_average | 0.16 | -0.27 | 0.56 | 0.49 | 0.92 |

| | | | | | | |
|---|---|---|---|---|---|---|
| fitbit_pa | BPI_current | 0.05 | -0.4 | 0.5 | 0.85 | 0.97 |
| fitbit_pa | FIQ_total | -0.11 | -0.54 | 0.37 | 0.64 | 0.97 |
| fitbit_pa | CSI | -0.01 | -0.46 | 0.47 | 0.97 | 0.99 |
| PSQI | PPT_average | 0.08 | -0.51 | 0.61 | 0.73 | 0.97 |
| PSQI | average_trap | 0.11 | -0.43 | 0.6 | 0.63 | 0.97 |
| PSQI | average_tib | -0.01 | -0.58 | 0.53 | 0.97 | 0.99 |
| PSQI | TS_final | -0.38 | -0.77 | 0.13 | 0.10 | 0.64 |
| PSQI | CPM_final | 0.43 | -0.09 | 0.82 | 0.06 | 0.60 |
| PSQI | BPI_worst | 0.09 | -0.44 | 0.58 | 0.71 | 0.97 |
| PSQI | BPI_least | -0.18 | -0.61 | 0.29 | 0.44 | 0.89 |
| PSQI | BPI_average | 0.10 | -0.41 | 0.59 | 0.66 | 0.97 |
| PSQI | BPI_current | -0.00 | -0.52 | 0.54 | 0.99 | 0.99 |
| PSQI | FIQ_total | 0.06 | -0.47 | 0.59 | 0.82 | 0.97 |
| PSQI | CSI | 0.21 | -0.32 | 0.67 | 0.37 | 0.89 |
| BMI | PPT_average | 0.28 | -0.21 | 0.67 | 0.23 | 0.88 |
| BMI | average_trap | 0.25 | -0.29 | 0.71 | 0.29 | 0.89 |
| BMI | average_tib | 0.21 | -0.29 | 0.63 | 0.37 | 0.89 |
| BMI | TS_final | 0.32 | -0.1 | 0.63 | 0.16 | 0.77 |
| BMI | CPM_final | -0.08 | -0.55 | 0.41 | 0.75 | 0.97 |
| BMI | BPI_worst | 0.45 | 0.07 | 0.73 | **0.05** | 0.60 |
| BMI | BPI_least | 0.36 | -0.08 | 0.71 | 0.12 | 0.66 |
| BMI | BPI_average | 0.31 | -0.12 | 0.65 | 0.18 | 0.77 |
| BMI | BPI_current | 0.49 | 0.13 | 0.7 | **0.03** | 0.55 |
| BMI | FIQ_total | 0.48 | 0.13 | 0.72 | **0.03** | 0.55 |
| BMI | CSI | 0.39 | -0.05 | 0.71 | 0.09 | 0.64 |
| HRV | PPT_average | 0.19 | -0.33 | 0.64 | 0.42 | 0.89 |
| HRV | average_trap | 0.33 | -0.16 | 0.71 | 0.16 | 0.77 |
| HRV | average_tib | 0.15 | -0.39 | 0.62 | 0.54 | 0.95 |
| HRV | TS_final | 0.21 | -0.32 | 0.68 | 0.37 | 0.89 |
| HRV | CPM_final | -0.06 | -0.54 | 0.45 | 0.79 | 0.97 |
| HRV | BPI_worst | 0.06 | -0.38 | 0.51 | 0.81 | 0.97 |
| HRV | BPI_least | -0.10 | -0.56 | 0.39 | 0.66 | 0.97 |
| HRV | BPI_average | -0.06 | -0.53 | 0.43 | 0.79 | 0.97 |
| HRV | BPI_current | 0.07 | -0.35 | 0.52 | 0.75 | 0.97 |
| HRV | FIQ_total | -0.24 | -0.65 | 0.23 | 0.32 | 0.89 |
| HRV | CSI | -0.21 | -0.59 | 0.27 | 0.38 | 0.89 |
| PSS | PPT_average | -0.08 | -0.61 | 0.45 | 0.74 | 0.97 |
| PSS | average_trap | 0.02 | -0.53 | 0.53 | 0.95 | 0.99 |
| PSS | average_tib | -0.18 | -0.69 | 0.34 | 0.44 | 0.89 |
| PSS | TS_final | 0.16 | -0.36 | 0.6 | 0.51 | 0.94 |
| PSS | CPM_final | 0.24 | -0.23 | 0.63 | 0.31 | 0.89 |
| PSS | BPI_worst | 0.43 | 0 | 0.74 | 0.06 | 0.60 |
| PSS | BPI_least | 0.07 | -0.44 | 0.53 | 0.77 | 0.97 |
| PSS | BPI_average | 0.41 | -0.07 | 0.76 | 0.07 | 0.64 |
| PSS | BPI_current | 0.64 | 0.21 | 0.88 | **0.00** | 0.07 |
| PSS | FIQ_total | 0.68 | 0.24 | 0.93 | **0.00** | 0.06 |

| | | | | | | |
|---|---|---|---|---|---|---|
| PSS | CSI | 0.67 | 0.31 | 0.88 | **0.00** | 0.06 |

Values represent Spearman correlation coefficients (rho), bootstrap 95% confidence intervals (CI), unadjusted p-values, and Benjamini–Hochberg False Discovery Rate (FDR) adjusted p-values.

**Note:** There were no participant dropouts, and data and plasma samples were available for all 20 participants. However, quantifiable cytokine concentrations were available for all 20 participants for IL-6, 13 participants for IL-10, and 19 participants for TNF-α because some measurements were below the quantitative range of the respective assays.

**Abbreviations:** EDII, Empirical Dietary Inflammatory Index; UPF, Ultra-Processed Food; PA, Physical Activity; PPT, Pressure Pain Threshold; trap, Trapezius; tib, Tibialis; TS, Temporal Summation; CPM, Conditioned Pain Modulation; BPI, Brief Pain Inventory; FIQ, Fibromyalgia Impact Questionnaire; CSI, Central Sensitization Inventory.

**Supplementary Table S4:** Bivariate associations between lifestyle factors and inflammatory biomarkers

| Variable 1 | Variable 2 | Spearman rho | 95% CI lower | 95% CI upper | p value | FDR p-value |
|---|---|---|---|---|---|---|
| BMI | IL-10 | 0.57 | -0.12 | 0.93 | **0.04** | 0.51 |
| BMI | IL-6 | 0.18 | -0.32 | 0.6 | 0.46 | 0.70 |
| BMI | TNF-α | 0.05 | -0.49 | 0.54 | 0.85 | 0.88 |
| EDII | IL-10 | 0.47 | -0.16 | 0.87 | 0.10 | 0.70 |
| EDII | IL-6 | 0.46 | -0.02 | 0.79 | **0.04** | 0.51 |
| EDII | TNF-α | 0.22 | -0.3 | 0.67 | 0.36 | 0.70 |
| HRV | IL-10 | -0.26 | -0.74 | 0.38 | 0.40 | 0.70 |
| HRV | IL-6 | -0.34 | -0.74 | 0.17 | 0.15 | 0.70 |
| HRV | TNF-α | -0.14 | -0.53 | 0.29 | 0.58 | 0.77 |
| Physical activity | IL-10 | -0.22 | -0.73 | 0.42 | 0.46 | 0.70 |
| Physical activity | IL-6 | -0.24 | -0.66 | 0.24 | 0.31 | 0.70 |
| Physical activity | TNF-α | -0.09 | -0.63 | 0.47 | 0.71 | 0.85 |
| PSQI | IL-10 | 0.15 | -0.49 | 0.7 | 0.62 | 0.78 |
| PSQI | IL-6 | 0.24 | -0.21 | 0.61 | 0.32 | 0.70 |
| PSQI | TNF-α | 0.16 | -0.35 | 0.61 | 0.52 | 0.73 |
| PSS | IL-10 | 0.08 | -0.55 | 0.64 | 0.78 | 0.86 |
| PSS | IL-6 | 0.19 | -0.29 | 0.61 | 0.43 | 0.70 |
| PSS | TNF-α | 0.07 | -0.4 | 0.51 | 0.79 | 0.86 |
| Sleep duration | IL-10 | -0.32 | -0.76 | 0.38 | 0.29 | 0.70 |
| Sleep duration | IL-6 | -0.2 | -0.68 | 0.33 | 0.39 | 0.70 |
| Sleep duration | TNF-α | 0.18 | -0.39 | 0.69 | 0.46 | 0.70 |
| UPF | IL-10 | -0.31 | -0.71 | 0.23 | 0.31 | 0.70 |
| UPF | IL-6 | 0.19 | -0.37 | 0.73 | 0.42 | 0.70 |
| UPF | TNF-α | 0.04 | -0.49 | 0.51 | 0.88 | 0.88 |

Values represent Spearman correlation coefficients (rho), bootstrap 95% confidence intervals (CI), unadjusted p-values, and Benjamini–Hochberg False Discovery Rate (FDR) adjusted p-values.

**Note:** There were no participant dropouts, and data and plasma samples were available for all 20 participants. However, quantifiable cytokine concentrations were available for all 20 participants for IL-6, 13 participants for IL-10, and 19 participants for TNF-α because some measurements were below the quantitative range of the respective assays.

**Abbreviations:** EDII, Empirical Dietary Inflammatory Index; UPF, Ultra-Processed Food; PA, Physical Activity; PSQI, Pittsburgh Sleep Quality Index; BMI, Body Mass Index; HRV, Heart Rate Variability; PSS, Perceived Stress Scale; IL-6, Interleukin-6; TNFA, Tumor Necrosis Factor-alpha; IL-10, Interleukin-10

**Supplementary Table S5:** Bivariate associations between inflammatory biomarkers and pain outcomes

| Variable 1 | Variable 2 | Spearman rho | 95% CI lower | 95% CI upper | p value | FDR p-value |
|---|---|---|---|---|---|---|
| IL6 | PPT_average | 0.07 | -0.43 | 0.54 | 0.77 | 0.959 |
| IL6 | average_trap | 0.04 | -0.49 | 0.49 | 0.85 | 0.959 |
| IL6 | average_tib | -0.04 | -0.54 | 0.44 | 0.87 | 0.959 |
| IL6 | TS_final | -0.10 | -0.47 | 0.32 | 0.68 | 0.959 |
| IL6 | CPM_final | 0.29 | -0.25 | 0.69 | 0.21 | 0.959 |
| IL6 | BPI_worst | -0.16 | -0.62 | 0.35 | 0.49 | 0.959 |
| IL6 | BPI_least | -0.16 | -0.68 | 0.33 | 0.50 | 0.959 |
| IL6 | BPI_average | -0.24 | -0.7 | 0.3 | 0.31 | 0.959 |
| IL6 | BPI_current | -0.07 | -0.59 | 0.44 | 0.77 | 0.959 |
| IL6 | FIQ_total | 0.08 | -0.45 | 0.63 | 0.73 | 0.959 |
| IL6 | CSI | 0.26 | -0.27 | 0.7 | 0.28 | 0.959 |
| TNFA | PPT_average | -0.55 | -0.79 | -0.16 | **0.02** | 0.2524 |
| TNFA | average_trap | -0.38 | -0.72 | 0.1 | 0.11 | 0.959 |
| TNFA | average_tib | -0.57 | -0.81 | -0.19 | **0.01** | 0.2524 |
| TNFA | TS_final | -0.34 | -0.68 | 0.12 | 0.16 | 0.959 |
| TNFA | CPM_final | -0.26 | -0.69 | 0.28 | 0.29 | 0.959 |
| TNFA | BPI_worst | 0.09 | -0.42 | 0.56 | 0.70 | 0.959 |
| TNFA | BPI_least | -0.01 | -0.54 | 0.52 | 0.98 | 0.9828 |
| TNFA | BPI_average | 0.01 | -0.47 | 0.49 | 0.96 | 0.9828 |
| TNFA | BPI_current | -0.08 | -0.58 | 0.44 | 0.74 | 0.959 |
| TNFA | FIQ_total | 0.08 | -0.43 | 0.53 | 0.73 | 0.959 |
| TNFA | CSI | 0.16 | -0.34 | 0.59 | 0.50 | 0.959 |
| IL10 | PPT_average | 0.07 | -0.58 | 0.6 | 0.82 | 0.959 |
| IL10 | average_trap | 0.25 | -0.42 | 0.76 | 0.41 | 0.959 |
| IL10 | average_tib | -0.12 | -0.7 | 0.5 | 0.71 | 0.959 |
| IL10 | TS_final | -0.12 | -0.7 | 0.52 | 0.69 | 0.959 |
| IL10 | CPM_final | -0.06 | -0.65 | 0.52 | 0.84 | 0.959 |
| IL10 | BPI_worst | 0.03 | -0.46 | 0.52 | 0.92 | 0.9783 |
| IL10 | BPI_least | 0.19 | -0.39 | 0.74 | 0.54 | 0.959 |
| IL10 | BPI_average | 0.16 | -0.37 | 0.63 | 0.60 | 0.959 |
| IL10 | BPI_current | 0.16 | -0.39 | 0.66 | 0.59 | 0.959 |
| IL10 | FIQ_total | 0.18 | -0.37 | 0.66 | 0.55 | 0.959 |
| IL10 | CSI | 0.39 | -0.2 | 0.78 | 0.18 | 0.959 |

Values represent Spearman correlation coefficients (rho), bootstrap 95% confidence intervals (CI), unadjusted p-values, and Benjamini–Hochberg False Discovery Rate (FDR) adjusted p-values.

**Note:** There were no participant dropouts, and data and plasma samples were available for all 20 participants. However, quantifiable cytokine concentrations were available for all 20 participants for IL-6, 13 participants for IL-10, and 19 participants for TNF-α because some measurements were below the quantitative range of the respective assays.

**Abbreviations:** IL-6, Interleukin-6; TNFA, Tumor Necrosis Factor-alpha; IL-10, Interleukin-10; PPT, Pressure Pain Threshold; trap, Trapezius; tib, Tibialis; TS, Temporal Summation; CPM, Conditioned Pain Modulation; BPI, Brief Pain Inventory; FIQ, Fibromyalgia Impact Questionnaire; CSI, Central Sensitization Inventory.
